# Bivalirudin anticoagulation for an infant with heparin resistance on ECMO: A case report

**DOI:** 10.1097/MD.0000000000039357

**Published:** 2024-10-11

**Authors:** Siqi Guo, Lan Chen, Jing Shi, Ge Zhang

**Affiliations:** a Key Laboratory of Birth Defects and Related Diseases of Women and Children (Sichuan University), Ministry of Education, Chengdu, Sichuan Province, China; b Department of Laboratory Medicine, West China Second University Hospital, Sichuan University, Chengdu, Sichuan Province, China; c Department of Neonatology, West China Second University Hospital, Sichuan University, Chengdu, China.

**Keywords:** anticoagulation, heparin resistance, infant

## Abstract

**Rationale:**

Extracorporeal membrane oxygenation (ECMO) technology in the field of intense care for children in China has developed rapidly, and it has become a key strategy for the rescue treatment of critically ill children and an advanced extracorporeal life support system. Compared with adults and children, neonatal respiratory disease with ECMO support has the best prognosis, with an average survival rate of 74%. Bleeding and thrombotic events during ECMO are common, morbid, and potentially lethal. Therefore, how to balance the coagulation state is the key to ECMO management.

**Patient concerns:**

A full-term male infant (2h 5min) was hospitalized for respiratory distress and cyanosis. With a history of premature rupture of membranes (>7 hours) and a birth weight of 3000 g, the patient had Apgar scores of 7, 8, and 9 at 1, 5, and 10 minutes, respectively.

**Diagnoses:**

This infant has the indication of extracorporeal membrane lung support. After full communication, venoarterial-ECMO was performed, and intravenous infusion of heparin was used for anticoagulation management.

**Interventions:**

We encountered an unreliable heparin monitoring in an infant on ECMO, which considered as heparin resistance. Subsequently, we switched the anticoagulant from heparin to bivalirudin and managed by using multiple laboratory tests including activated clotting time (ACT) and activated partial thromboplastin time. The phenomenon of inconsistent monitoring results occurred later. To help the clinic to adjust the anticoagulation dose accurately, we adopted additional tests such as thrombin-antithrombin complex (TAT) and fibrin/fibrinogen degradation products and applied comparison of thrombela stogram (TEG)-ACT with anticoagulated specimens and bedside non-anticoagulated ACT, then recommended clinicians to use activated partial thromboplastin time combined with TAT.

**Outcomes:**

In collaboration with other symptomatic supportive treatments, the ECMO flow was gradually reduced, the respiratory and circulatory functions were stable after reducing the flow rate, there was no bleeding tendency, and the ECMO was finally evacuated.

**Lessons:**

Due to the unique physiological characteristics of newborns, the hemostatic changes differ significantly from those in adults. Precise monitoring of anticoagulation becomes a critical and challenging task. Bivalirudin can be effectively used for anticoagulation management in neonatal ECMO; however, due to its unique characteristics, precise dose adjustment poses a challenge. Selecting the optimal laboratory monitoring indicators is crucial in this regard. In some cases, bedside ACT may not be the optimal anticoagulation monitoring parameter, and when necessary, comparative analysis can be conducted using anticoagulant-sample ACTs such as thrombela stogram-ACT. Traditional markers such as D-dimer/fibrinogen degradation products and newer indicators like TAT can reflect the activation of coagulation and assist in monitoring the anticoagulation effect, especially when there is conflicting information among the monitoring parameters.

## 1. Introduction

In recent decades, extracorporeal membrane oxygenation (ECMO) technology in the field of intensive care for children in China has developed rapidly, and it has become a key strategy for the rescue treatment of critically ill children and an advanced extracorporeal life support system.^[1]^ Compared with adults and children, neonatal respiratory disease with ECMO support has the best prognosis, with an average survival rate of 74%.^[2]^ Bleeding and thrombotic events during ECMO are common, morbid, and potentially lethal. Therefore, how to balance the coagulation state is the key to ECMO management. Continuous infusion of heparin is the first choice for ECMO anticoagulation, which should be used individually in the clinic. Activated clotting time (ACT), activated partial thromboplastin time (APTT), and anti-factor Xa (anti-Xa) are the preferred anticoagulation monitoring indicators. The anticoagulant effect of heparin is affected by the activity level of antithrombin III (AT-III) in the body (80%–120%). Infants with low AT-III are prone to heparin resistance. When heparin resistance or heparin-induced thrombocytopenia (HIT) occurs, instant adjustment of anticoagulation is needed. Bivalirudin, a direct thrombin inhibitor that inhibits reversibly binds thrombin without the cofactor AT-III. It has been used successfully for anticoagulation in patients on ECMO. However, it was used unconventionally in the neonatal population. There are few published data regarding bivalirudin anticoagulation for neonates on ECMO and no consistent coagulation monitoring protocol in the literature to date.^[3,4]^

We encountered unreliable heparin monitoring in an infant on ECMO, which was considered heparin resistance. Subsequently, we switched the anticoagulant from heparin to bivalirudin and managed it by using multiple laboratory tests, including ACT and APTT. However, the standardized management of bivalirudin administration in infant ECMO patients with heparin resistance has not been clearly elucidated due to limited data. The phenomenon of inconsistent monitoring results between ACT and APTT occurred later. To help clinicians adjust the anticoagulation dose accurately, we adopted additional tests, such as thrombin–antithrombin complex (TAT) and fibrin/fibrinogen degradation products (FDPs), and compared thrombela stogram (TEG)-ACT with anticoagulated specimens and bedside nonanticoagulated ACT. Then, we recommended that clinicians use APTT combined with TAT, successfully maintain the clinical efficacy and safety of bivalirudin anticoagulation therapy during ECMO, and finally help the patient achieve weaning.

## 2. Case

### 2.1. Patient information

A 2-hour and 5-minute male neonate was admitted to the hospital due to shortness of breath and cyanosis. The patient was G1P1, gestational age 38 + 4 weeks, delivered vaginally, premature rupture of membranes for more than 7 hours, birth weight 3000 g, Apgar score at 1-5-10 minutes was 7 (skin color, cry, muscle tension 1 point)-8 (1 point deducted for crying and muscle tension)-9 (1 point deducted for muscle tension). Tracheal intubation, high-frequency ventilator-assisted ventilation, repeated pulmonary surfactant supplementation, continuous nitric oxide inhalation, enhanced anti-infection, circulatory support, and other treatments were performed, but the patient’s oxygen saturation remained poor. The patient has experienced a progressive decrease in heart rate and oxygen saturation, severe internal environment disturbance, aggravated air leakage, repeated fever, decreased urination, and coagulation disorders.

### 2.2. Diagnostic assessment

Doctors actively performed cardiopulmonary resuscitation, thoracentesis and closed drainage, acid correction and volume expansion to correct the patient’s internal environment disorders, used various drugs to lower pulmonary artery pressure, infused various blood products to correct coagulation function, diuresis, protection of organs, etc. The heart rate and blood pressure were maintained, but the patient developed hypoxemia (partial pressure of oxygen [PaO_2_ <40 mm Hg]), and the oxygenation index continued to be >40 and was combined with multiple organ failure. This infant has the indication of extracorporeal membrane lung support. After full communication with family members, venoarterial-ECMO was performed, and intravenous infusion of heparin was used for anticoagulation management.

### 2.3. Therapeutic intervention

Heparin was commenced at 10 µg/kg/h on cannulation for ECMO. The target ranges of heparin anticoagulation were ACT 180 to 220 seconds, APTT 1.5 to 2.5 times the baseline APTT (41.7–69.5 seconds),^[5,6]^ and heparin anti-Xa 0.35–0.7 U/mL. Strict anticoagulation management followed. ACT was performed every 1 to 2 hours, PT/APTT/Fbg/DD/FDP/ATIII was examined every 8 hours, and heparin anti-Xa was performed daily.

### 2.4. Follow-up and outcomes

**Figure 1.**
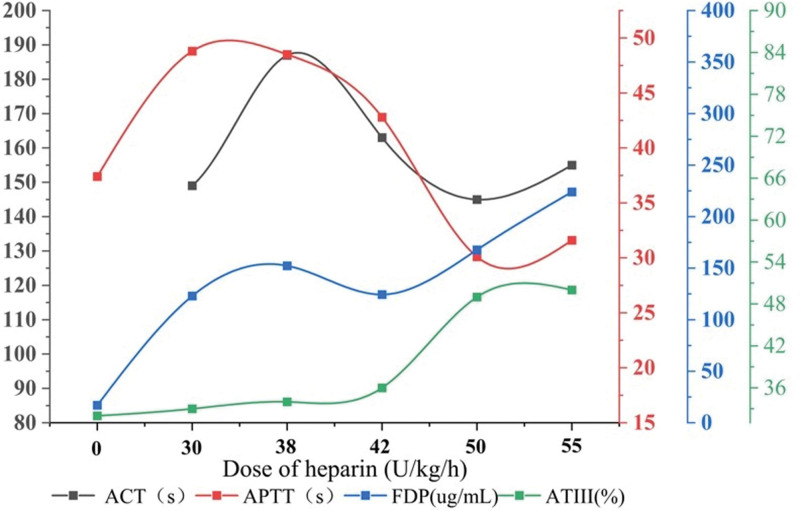
Heparin resistance (ACT and APTT did not increase with increasing doses of heparin, while FDP increased gradually, suggesting that heparin anticoagulation was not effective). ACT = activated clotting time, APTT = activated partial thromboplastin time, ATIII = antithrombin III, FDP = fibrinogen degradation products.

As the dose of heparin increased to 55 µg/kg/h, ACT and APTT did not prolong accordingly. Meanwhile, the anti-Xa levels were lower than 0.1 U/mL combined with D-Dimer >40 mg/L fibrinogen equivalent unit and TAT >120 ng/mL, indicating that the patient was in a hypercoagulated state. Considering that the main reason for the poor anticoagulation effect of neonatal heparin is the insufficient ATIII level (the initial ATIII level was 32%), the ATIII was supplemented by plasma infusion, but the anticoagulation index still fell far short of the control target (Fig. 1). Clinicians also gave feedback that a micro clot in the catheter was seen.

**Figure 2.**
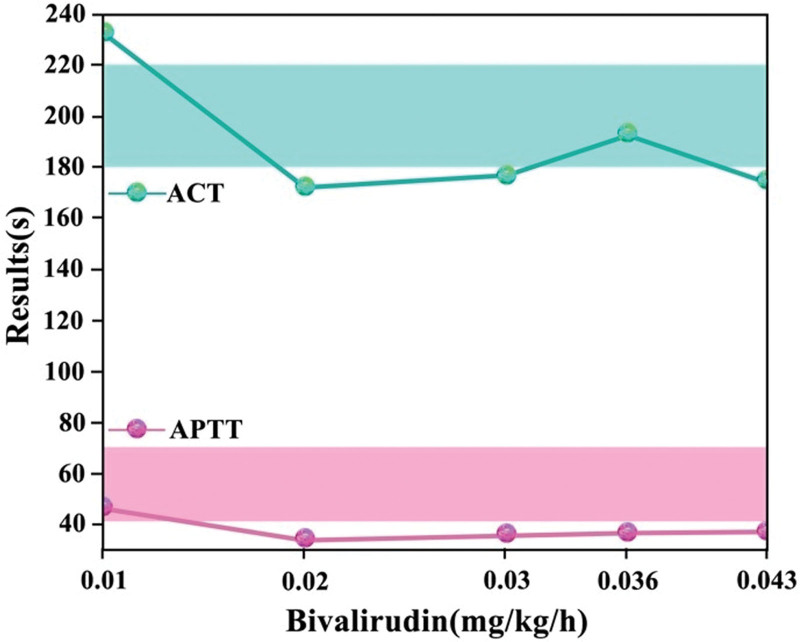
APTT and ACT are inconsistent during bivalirudin anticoagulation monitoring. ACT = activated clotting time, APTT = activated partial thromboplastin time.

Heparin resistance was considered, and the cause of resistance was unknown. After consultation with the pharmacy department, clinicians decided to replace heparin and use bivalirudin for anticoagulation. Due to the lack of guidelines and consensus on the application of bivalirudin anticoagulation in neonatal ECMO, clinicians and laboratories were only able to make protocol adjustments based on the limited literature.^[7–9]^ Anticoagulation was changed to bivalirudin with a starting dose of 0.1 mg/kg/h and a maintained dose at 0.01 mg/kg/h 2 hours later. The coagulation indexes improved significantly on the same day, with an APTT of 56.9 seconds and an ACT of 220 seconds. However, during subsequent anticoagulation monitoring, the APTT was significantly shortened, suggesting inadequate anticoagulation, while the bedside ACT fluctuated within the target value (Fig. 2).

Anticoagulation monitoring based on which indicators became a new challenge. The laboratory ran TAT combined with FDP to identify the patient’s coagulation status and found that the TAT was 68.6 ng/mL, and the FDP was >200 mg/dL. Meanwhile, laboratories used TEG-ACT with anticoagulated specimens to compare with bedside nonanticoagulated ACT. The anticoagulation TEG-ACT was found to be significantly short, which was consistent with APTT. Hence, the laboratories proposed the use of APTT rather than bedside ACT for bivalirudin dose adjustment and suggested the inclusion of TAT as an additional indicator. The new protocol for coagulation monitoring was adjusted. The APTT reached the desired goal (50–60 seconds) when the final bivalirudin maintenance dose was gradually adjusted to near 0.178 mg/kg/h. In collaboration with other symptomatic supportive treatments, the patient’s pneumothorax was absorbed, no gas was seen in the closed chest drainage bottle, the blood gas analysis was normal for lactate, the lung condition improved significantly, the ECMO flow was gradually reduced, the respiratory and circulatory functions were stable after reducing the flow rate, there was no bleeding tendency, and the ECMO was finally evacuated.

In this particular case, we have employed a mode of simultaneous monitoring using multiple indicators. During this process, noticeable inconsistencies between the indicators emerged, requiring further experimentation and comprehensive clinical assessment. This situation vividly underscores the difficulties and challenges associated with anticoagulation management for newborns. This has led to the development of an anticoagulation management program.

## 3. Discussion

This is the first report of a Chinese infant on ECMO who switched to bivalirudin anticoagulation after the occurrence of heparin resistance. The occurrence of heparin resistance is the reason in this case that compelled us to seek the use of bivalirudin as an alternative anticoagulant. It is defined as the inability of an adequate dose of heparin to achieve a desired ACT or other monitoring indicators.^[10]^ Underlying causes of heparin resistance can be complex and are usually multifactorial; the most prominent mechanisms include antithrombin deficiency (acquired or congenital), especially for the newborn, and congenital antithrombin deficiency is the primary cause.^[11,12]^ This is also why we initially aimed to correct heparin resistance through plasma transfusion and increasing heparin dosage. However, to our surprise, even when heparin reached 55 µg/kg/h and AT reached nearly 50%, resistance still existed, and the high TAT and DD/FDP levels also supported this conclusion. Additionally, we do not believe that some common causes of heparin resistance, such as inflammation, low albumin concentrations and elevated platelet levels, can reasonably explain the refractory heparin resistance observed in this case. Due to the urgency of this situation, we were unable to further investigate the underlying causes of heparin resistance, such as conducting genetic screening. At this point, switching anticoagulants became our primary choice.

Bivalirudin is a direct thrombin inhibitor, a promising alternative anticoagulant for pediatric ECMO patients who have failed unfractionated heparin. However, the use of bivalirudin in infants is relatively rare. The reason for the use of bivalirudin varied, including heparin resistance, unstable ACTs, clotting on heparin, and HIT.^[7,13]^ Infants may require higher bivalirudin doses, and they have more rapid clearance of bivalirudin than older children.^[14]^ Since Pollak et al^[15]^ reported the use of bivalirudin in a 5-day-old infant with HIT. Snyder et al^[3]^ described 1 perioperative anticoagulation protocol for 42 neonates with CDH treated with ECMO. Bivalirudin appears to be safe and effective. However, there remain many unanswered questions, and further controlled studies should be performed. Close monitoring by clinicians and laboratories is recommended.

ACT and APTT are the preferred anticoagulant monitoring indicators for ECMO anticoagulation, and anti-Xa is also recommended.

ACT, as a bedside indicator, is indeed more convenient, but it is also more susceptible to variations in the activity of coagulation factors, platelet count and function, as well as environmental factors, primarily because it operates within a nonstandardized whole blood testing system. Consequently, the instructions for the bedside ACT that we utilize specify that in the event of any result uncertainty, alternative methods or a retest using anticoagulated blood ACT should be considered. This is precisely why, in this particular case, we opted for TEG-ACT with anticoagulated blood for the retest.

As a reliable indicator, APTT demonstrates a strong correlation with low heparin concentrations. Employing APTT for monitoring necessitates the establishment of a reference range within the laboratory that is closely linked to heparin or other drug concentrations. However, due to notable divergences in APTT reagent formulations and between batches, this range should be adjusted accordingly. For infants, who exhibit a delicate balance between reduced coagulation and anticoagulation and manifest significant individual disparities, the standard APTT levels vary considerably. Consequently, determining a target range becomes a challenging task. Despite certain studies proposing APTT target ranges, the lack of reagent standardization and the presence of substantial individual differences make it less practical, especially for premature newborns. Therefore, employing APTT as an indicator for anticoagulant therapy in this context is not particularly convenient. In our case, considering the characteristics of both the reagents and the patients, we set the monitoring range for APTT to be between 50 and 60 seconds.

Anti-Xa is another precise indicator that directly reflects the efficacy of heparin anticoagulation. However, it shares a similar inconvenience with APTT—its monitoring target range needs to be meticulously assessed. Additionally, for newborns, there are notable challenges associated with anti-Xa testing. Apart from the expense and longer testing duration, the necessity for a larger sample volume stands out as a significant drawback. The risk of iatrogenic anemia in newborns makes frequent monitoring impractical. Last, it is important to note that due to differing target sites of action, anti-X testing cannot be employed to monitor bivalirudin.

As mentioned earlier, despite the availability of so many monitoring indicators, the lack of a consistent coagulation monitoring protocol is very troubling. Among these indicators, there is no single indicator that is flawless. Hence, in this particular case, we have also employed a mode of simultaneous monitoring using multiple indicators. During this process, noticeable inconsistencies between the indicators emerged, requiring further experimentation and comprehensive clinical assessment. This situation vividly underscores the difficulties and challenges associated with anticoagulation management for newborns.

In this case, we encountered inconsistent monitoring results between the bedside ACT and APTT. Achieving the target ACT and dealing with significantly lower APTT levels than standard presented challenges in adjusting the bivalirudin dosage clinically. To address this issue, we introduced additional monitoring indicators, primarily DD/FDP and TAT. DD/FDP, as major fibrin degradation products, exhibited elevated levels, indicating active fibrinolysis. TAT, serving as a marker for coagulation activation, typically maintains very low levels in normal conditions, only rising during coagulation initiation. This reflects early coagulation abnormalities and thrombosis risk.^[16,17]^ By incorporating TAT, a more sensitive and earlier indicator, we could more accurately determine the body’s actual coagulation balance. Both the high DD/FDP and TAT values suggested the presence of hypercoagulability, indicating that relying solely on bedside ACT results was unreliable. Consequently, we introduced TEG-ACT, and eventually, we decided to adjust the dosage according to APTT. Subsequent clinical feedback also supported our chosen approach. This also suggests that for complex cases, the mutual validation of multiple laboratory parameters may be more conducive to making accurate assessments.

## 4. Conclusion

Due to the unique physiological characteristics of newborns, hemostatic changes differ significantly from those in adults. Precise monitoring of anticoagulation becomes a critical and challenging task. In this case, 2 issues were highlighted: first, the recognition and management of heparin resistance in ECMO. Second, the solution of inconsistency between anticoagulation monitoring indicators. When faced with important clinical decisions, comprehensive assessment of multiple indicators is an effective means to avoid adverse consequences caused by test errors, and TAT is expected to become one of the effective indicators for anticoagulation monitoring in the future.

There may still be some limitations in this study. First, newborns are a special population, although there is some literature on the ECMO therapy, there is still a lack of reference for this rare condition in the process of coagulation management, which may lead to an imperfect discussion. Second, after ruling out antithrombin deficiency, what exactly causes heparin resistance? Is it endothelial cells, platelets or inflammation? Due to the patient’s critical and urgent condition, we did not explore this in detail at that time, but went straight to the anticoagulant replacement protocol. This is a question we need to explore further in depth, and we will observe and accumulate experience in our future study.

## Author contributions

**Data curation:** Siqi Guo.

**Formal analysis:** Siqi Guo.

**Investigation:** Siqi Guo.

**Methodology:** Siqi Guo.

**Writing – original draft:** Siqi Guo.

**Conceptualization:** Jing Shi, Ge Zhang.

**Resources:** Jing Shi.

**Supervision:** Jing Shi, Ge Zhang.

**Validation:** Jing Shi, Ge Zhang.

**Visualization:** Ge Zhang.

**Writing – review & editing:** Ge Zhang.
